# The Association Between Technology Use and Health Status in a Chronic Obstructive Pulmonary Disease Cohort: Multi-Method Study

**DOI:** 10.2196/jmir.9382

**Published:** 2018-04-02

**Authors:** Matthew Witry, Alejandro Comellas, Jacob Simmering, Philip Polgreen

**Affiliations:** ^1^ Department of Pharmacy Practice and Science College of Pharmacy University of Iowa Iowa City, IA United States; ^2^ Department of Internal Medicine Carver College of Medicine University of Iowa Iowa City, IA United States; ^3^ Signal Center for Health Innovation University of Iowa Iowa City, IA United States

**Keywords:** pulmonary disease, chronic obstructive, telemedicine, wireless technology, electronic mail, remote consultation, patient simulation, surveys

## Abstract

**Background:**

Telemedicine and electronic health (eHealth) interventions have been proposed to improve management of chronic obstructive pulmonary disease (COPD) for patients between traditional clinic and hospital visits to reduce complications. However, the effectiveness of such interventions may depend on patients’ comfort with technology.

**Objective:**

The aim was to describe the relationship between patient demographics and COPD disease severity and the use of communication-related technology.

**Methods:**

We administered a structured survey about the use of communication technologies to a cohort of persons in the COPDGene study at one midwestern hospital in the United States. Survey results were combined with clinical and demographic data previously collected as part of the cohort study. A subsample of patients also completed eHealth simulation tasks. We used logistic or linear regression to determine the relationship between patient demographics and COPD disease severity and reported use of communication-related technology and the results from our simulated eHealth-related tasks.

**Results:**

A total of 686 patients completed the survey and 100 participated in the eHealth simulation. Overall, those who reported using communication technology were younger (*P*=.005) and had higher incomes (*P*=.03). Men appeared less likely to engage in text messaging (*P*<.001) than women. Patients who spent more time on tasks in the eHealth simulation had greater odds of a COPD Assessment Test score >10 (*P*=.02) and walked shorter distances in their 6-minute walk tests (*P*=.003) than those who took less time.

**Conclusions:**

Older patients, patients with lower incomes, and less healthy patients were less likely to report using communication technology, and they did not perform as well on our simulated eHealth tasks. Thus, eHealth-based interventions may not be as effective in these populations, and additional training in communication technology may be needed.

## Introduction

Chronic obstructive pulmonary disease (COPD) is the third-leading cause of death in the United States and is a major cause of morbidity and mortality [1]. The management of COPD includes smoking cessation, pulmonary rehabilitation, oxygen, and pharmacologic treatments such as systemic steroids, inhalers, and periodic antibiotic treatments [2]. Diligent adherence to these therapies is associated with decreased mortality, fewer exacerbations and hospitalizations, and quality of life improvements [3-5]. Most patients, however, are nonadherent to approximately half of their therapies [6]. Barriers to COPD therapy adherence include the complexity and cost of regimens, incorrect medication knowledge and beliefs, health literacy, and cognitive deficits [4,7,8]. The management of COPD can be especially challenging in rural areas where access to care is limited. Indeed, rural patients with COPD have been found to have worse outcomes [9].

One approach for supporting patient self-management between traditional office visits, including in rural areas, has been to use telephone-based interventions, an approach often referred to as “telehealth.” Such programs have used nurses or other members of the health care team to regularly monitor patient symptoms via telephone. When patient worsening is identified, actions such as the early prescribing of antibiotics or steroids can be administered [10]. However, the results of telehealth interventions are mixed [10,11].

Recently, cellular and internet-connected devices have been suggested as an alternative or extension to telehealth interventions as a way for patients and providers to engage in bidirectional electronic information exchange. These interventions have been referred to as *electronic health (eHealth)* or included under the umbrella of telehealth. For example, internet-connected devices have been used as a means to measure a patient’s lung function and oxygen saturation, to provide information about a patient’s clinical condition to clinicians, or provide direct recommendations to the patient [12]. Identifying interventions that increase the quality of patient-provider communication, symptom reporting, and data exchange are critical as health care systems look for strategies to decrease COPD exacerbations and hospital admissions [13]. However, studies on internet-based telehealth interventions in patients with COPD have reported mixed results as well [14-17].

Although the use of the internet among older adults is increasing, it still is lower than among younger populations, with rates of internet adoption at 58% for persons aged 65 years and older [18]. A study examining the use of secure messaging between patients and physicians found one-third of those aged 55 years and older had used secure messaging, but there were significant disparities in use due to income, race, education, and health status [19]. Barriers to internet and information technology adoption for older adults include lack of broadband access, rural living, lower ownership of internet-enabled tablets or mobile phones, physical disabilities (ie, impaired vision), the preference for assistance when interacting with a new device, and health literacy [20-23]. Patients with COPD may also have a lower level of internet use because advanced age is a risk factor for COPD.

This paper aims to report the relationship between COPD and ownership and use of various technologies and devices. Specifically, we examine the effect of COPD severity and patient demographics on not only technology use and ownership, but also completion time for a simulated eHealth task.

## Methods

### Overview

All data were collected from adults participating in the COPDGene multicenter cohort study at the University of Iowa Hospitals and Clinics. The COPDGene study details have been reported elsewhere [24]. Briefly, COPDGene is a multicenter observational study including current and former smokers designed to identify genetic factors associated with COPD. A total of 10,192 non-Hispanic white and African American adults aged 45 to 80 years with a minimum 10 pack-year smoking history were enrolled between January 2008 and April 2011. Participants were phenotyped by completing questionnaires, blood tests, imaging, and spirometry. During a scheduled COPDGene study visit, patients were surveyed using a structured interview conducted by a respiratory therapist research nurse either by telephone or in person. Surveys were completed between August 2014 and June 2016. Beginning in May 2015, interested patients also could participate in a brief simulation in which a set of simple eHealth tasks were performed and timed. Patients were compensated for taking part in the COPDGene cohort, but no additional compensation was provided for taking part in the survey. Those agreeing to participate in the additional eHealth simulation were offered a US $10 gift card for their time. Both the survey and eHealth simulation substudy were approved by the COPDGene group and the University of Iowa Institutional Review Board. This study was conducted in accordance with the amended Declaration of Helsinki.

### Survey

The survey was administered via a structured interview. The objective was to assess the use of various communication technologies that could be used in eHealth interventions including cellular telephones, text messaging, email, and video chat (Multimedia Appendix 1). A combination of yes/no questions and several short responses were used. Interviews lasted 5 to 10 minutes and responses were recorded in RedCap (Nashville, TN, USA), a secure online database hosted at the University of Iowa and linked to the patient’s COPDGene identifier.

### Clinical Data

Survey data were linked to clinical and demographic data from the COPDGene study database using the study identifier. Variables included age, gender, income level, and validated measures of disease progression—the 6-minute walk test and COPD Assessment Test (CAT) [25] score. These variables were collected during their most recent COPDGene visit.

### eHealth Simulation

Six months after the structured interviews began, we invited participants to engage in a series of timed eHealth simulation exercises. These tasks involved launching an app, entering an access code, and responding to two CAT items. Participants performed these tasks on a laptop computer running Microsoft Windows (Redmond, WA, USA) and two tablet computers, one running the iOS mobile operating system (Cupertino, CA, USA) and one running the Android operating system (Mountain View, CA, USA). The time required for study participants to perform each task was recorded using a digital stopwatch. The order of devices was randomized to allow for comparisons.

### Analysis

To describe technology ownership and use, we calculated the percentages of ownership of computers and cell phones, as well as the use of email and video chat services, such as Skype (Redmond, WA, USA). For patients who owned a cell phone, we computed percentages of those who carry a phone regularly, own an internet-enabled mobile phone (smartphone), and use text messaging. We also calculated the percentages of those who use text messaging, given they either own a smartphone or use a cell phone regularly.

To determine if technology use differed by demographic characteristics, we estimated six logistic regression models. All models had age, sex, and income as covariates, and the outcome variables were cell phone ownership, smartphone ownership, computer ownership, use of text messaging, use of email, and use of video chat.

To determine if technology use differed by the severity of the patient’s disease, we estimated three sets of models. The outcomes for each set were the result of the 6-minute walk test and dichotomized CAT score (<10, ≥10). Because the result of the 6-minute walk test is continuous, we estimated linear regression models. For the dichotomous CAT score variable, we estimated logistic regression models. For each set of models, we estimated three separate models (one for each technology): text messaging, email, and video chat. Covariates for all models were patient age, sex, income, and a measure of technology use.

We characterized technology use in three ways. First, we divided ownership and use into three levels: nonowner of relevant technology, owner/nonuser of relevant technology, and owner/user of relevant technology. Second, we considered standardized task time from the eHealth simulations. We first converted each task time into a *z* score and took the sum of the normalized task times for the use of the iPad, Android tablet, cell phone texting, and email as a direct measure of familiarity of comfort with the given technology.

Finally, we considered the ability of simple questions about technology ownership and use (eg, “Do you own a smartphone, computer, or tablet?” and the video chat measure “Do you use Skype?”) to predict task performance. We estimated 12 linear regression models, one for each combination of device tested (laptop, Android tablet, iPad, and cell phone) and task (email, text messaging, and video chat). The outcome was task time, and the covariates were gender, age income, and a variable representing the use/nonuse of that task (nonowner, owner, nonuser, and user).

All statistical analysis was completed using R 3.2 (R Foundation for Statistical Computing, Vienna, Austria).

## Results

### Survey Results

There were 712 persons approached to complete the structured interview survey, of these 686 (96.3%) participated and provided complete survey data. In all, 100 patients also participated in the eHealth simulation task subsample out of 256 who were approached (39.1%). Demographics and summary statistics of the full sample and the subsample who performed the eHealth simulation tasks can be found in Table 1. The mean age of participants who completed the survey was 68.7 (SD 8.2) years, and 52.2% (358/686) were female.

**Table 1:** Characteristics of study participants.

Characteristic		Study sample (N=686)	Task subsample (n=100)
Age (years), mean (SD)		68.7 (8.2)	65.6 (7.8)
Gender (female), n (%)		358 (52.2)	66 (66.0)
**Income (US $), n (%)**			
	<15,000	42 (6.1)	8 (8.0)
	15,000-35,000	154 (22.4)	22 (22.0)
	35,000-50,000	139 (20.3)	17 (17.0)
	50,000-75,000	160 (23.3)	23 (23.0)
	>75,000	133 (19.4)	18 (18.0)
	Missing	58 (8.5)	12 (12.0)
CAT^a^ Score, mean (SD)		9.6 (6.9)	9.8 (7.8)
Six-Minute Walk, mean (SD)		1410.3 (402.7)	1442.5 (367.3)

^a^CAT: COPD Assessment Test.

**Table 2:** Description of technology ownership and use for the participants and mean task time for the subsample of participants engaging in the eHealth simulation tasks (N=686).

Technology use characteristic		Participants
Cell phone ownership, n (%)		645 (94.0)
**Cell phone owners who..., n (%)**		
	Have a smartphone	307 (47.6)
	Carry their phone daily	510 (79.1)
	Send/receive text messages	442 (68.5)
	Send/receive text messages given ownership of a smartphone	286 (93.2)
	Send/receive text messages given daily carrying of a cell phone	384 (75.3)
Computer ownership, n (%)		607 (88.8)
Use email given computer ownership, n (%)		564 (92.9)
Use video chat (Skype), n (%)		195 (32.1)
**Task times (simulation subsample only), mean (SD)**		
	iPad	76.3 (46.9)
	Android	49.4 (30.6)
	Laptop	52.8 (25.9)
	Basic phone texting	55.0 (26.4)

**Table 3:** Logistic model of demographic predictors of ownership and use for the total sample.

Characteristic		Own cell phone		Own smartphone		Use text messaging		Own computer		Use email		Use video chat	
		OR (95% CI)	*P*	OR (95% CI)	*P*	OR (95% CI)	*P*	OR (95% CI)	*P*	OR (95% CI)	*P*	OR (95% CI)	*P*
Age^a^		0.89 (0.84-0.94)	<.001	0.92 (0.90-0.94)	<.001	0.87 (0.84-0.89)	<.001	0.95 (0.92-0.99)	.005	0.93 (0.89-0.97)	.002	0.99 (0.96-1.01)	.20
Male^b^		0.54 (0.26-1.10 )	.08	0.81 (0.57-1.16)	.24	0.66 (0.45-0.98)	<.001	0.96 (0.57-1.60)	.87	0.59 (0.31-1.12)	.10	0.82 (0.57-1.18)	.27
**Income (US $1000)**															
	<15	0.67 (0.19-2.37)	.53	0.86 (0.38-1.91)	.70	0.87 (0.36-2.12)	.76	0.41 (0.18-0.95)	.04	0.91 (0.23-3.57)	.89	0.98 (0.39-2.46)	.96
	15-35	1.00		1.00		1.00		1.00		1.00		1.00	
	35-50	1.04 (0.44-2.44)	.93	1.47 (0.86-2.51)	.15	0.94 (0.53-1.64)	.81	1.42 (0.71-2.85)	.31	1.51 (0.53-3.63)	.35	1.24 (0.69-2.22)	.46
	50-75	3.19 (0.98-10.45)	.05	1.63 (0.98-2.72)	.05	1.31 (0.75-2.28)	.34	2.06 (0.97-4.40)	.06	1.50 (0.63-3.53)	.35	1.68 (0.97-2.91)	.06
	>75	5.99 (1.26-28.46)	.02	3.29 (1.92-5.68)	<.001	1.95 (1.06-3.60)	.03	11.27 (2.52-50.36)	.001	8.16 (1.74-38.21)	.007	2.95 (1.70-5.14)	<.001
	Declined	0.67 (0.19-2.37)	.31	0.71 (0.34-1.46)	.34	0.88 (0.43-1.81)	.72	0.94 (0.41-2.16)	.89	1.08 (0.36-3.29)	.88	0.98 (0.44-2.20)	.97

^a^Age is continuous.

^b^Male is in comparison to female.

A description of technology ownership and use is found in Table 2. Nearly all (94.0%, 645/686) participants owned a cell phone, and most (74.3%, 510/686) of those carried their phone daily. Somewhat fewer (89%, 607/686) participants owned a computer, although nearly all (92.9%, 564/607) computer owners used email. A third (195/607) used video chat. The mean length of time needed for the eHealth simulations ranged from 49.4 (SD 30.6) seconds for the Android tablet to 76.3 (SD 46.9) seconds for the iOS tablet. Demographic predictors of technology use are summarized in Table 3. Generally, increasing age was associated with lower odds of owning or using technology. Also, a high income (greater than US $75,000/year) had higher odds of owning and using technology compared to lower income groups. Notably, among cell phone owners, men had odds of texting that were 66% of those of women.

**Table 4:** Logistic and ordinary least squares models including standardized eHealth simulation task times predicting disease severity (n=100).

Characteristic		CAT score ≥10^a^		Distance walked^b^	
		OR (95% CI)	*P*	OR (95% CI)	*P*
Age		0.93 (0.87, 1.00)	.04	2.78 (–6.31, 11.87)	.54
Male		1.30 (0.45, 3.69)	.62	72.91 (–71.37, 217.19)	.32
**Income (US $)**					
	<15,000	5.10 (0.72, 36.11)	.10	–310.27 (–579.59, –40.95)	.02
	15,000-35,000	Reference		Reference	
	35,000-50,000	0.53 (0.11, 2.66)	.43	163.26 (–56.20, 382.73)	.14
	50,000-75,000	2.44 (0.63, 9.40)	.19	158.26 (–38.72, 355.24)	.11
	>75,000	0.45 (0.09, 2.33)	.33	113.80 (–96.61, 323.20)	.28
	Declined	3.49 (0.67, 18.22)	.13	–37.39 (–271.88, 197.10)	.75
Standardized task time		1.27 (1.04, 1.55)	.02	–40.78 (–67.41, –14.15)	.003

^a^CAT: COPD Assessment Test. CAT score is based on logistic model.

^b^The 6-minute walk is based on ordinary least squares model.

In all cases (text messaging, email, and video chat), being an owner and user was associated with a lower CAT score compared with owner/nonusers and nonowners (Multimedia Appendix 2). Multimedia Appendix 3 repeats the analysis of Multimedia Appendix 2 but considers the distance covered in a 6-minute walk instead of CAT score. A statistically significant increase in distance covered among owner/users relative to owner/nonusers of email and video chat was observed, but not for text messaging. Nonowners of a computer or a smartphone walked a longer distance than owner/nonusers of email. None of the other nonowners traveled a statistically significant distance relative to owner/nonusers.

### eHealth Simulation Results

Results of a regression of standardized task time from the eHealth simulation and disease severity are reported in Table 4. Increased task time was statistically significantly associated with having a CAT score greater than 10 and recording fewer steps on the 6-minute walk test, surrogate markers for increased disease severity. For each standard deviation from the mean task time, we found a 27% increase in the odds of having a CAT score of 10 or greater. Likewise, for each standard deviation increase from the mean task time, we found a 41-unit decrease in the distance covered in the 6-minute walk test.

Lastly, after adjustment for age, sex, and income, users of video chat took less time than owner/nonusers to complete the laptop, Android tablet, iOS, and text messaging tasks: OR 10.2 (95% CI –0.5 to –20.0), OR 6.3 (95% CI –16.5 to 3.9), OR 16.4 (95% CI –33.6 to 0.8), and OR 6.3 (95% CI –37.3 to 39.5) seconds, respectively. On the other hand, nonowners took more time than owner/nonusers on these tasks: OR 58.4 (95% CI 21.8-94.9), OR 42.7 (95% CI 4.5-80.8), OR 22.7 (95% CI –42.7 to 87.1), and OR 1.1 (95% CI –16.5 to 3.9) seconds, respectively. Lastly, in Multimedia Appendix 4, we report the results of models of task time from the eHealth simulation and reported ownership/nonownership and use/nonuse of the devices. Email users were significantly faster when using a laptop or Android tablet than nonusers of email. Similarly, video chat users were significantly faster on the laptop and iOS device than nonowners.

## Discussion

Our results showed that most respondents in our COPD cohort, regardless of income or age, had access to either a cell phone or personal computer, and most reported comfort using these devices. However, use and familiarity with newer technologies, such as smartphones or video chat, were less common. In general, we found that participants with more severe disease were less likely to report the use of technology. In addition, when we tested participants’ ability to use technology in a simulated eHealth task, we found that patients who had more difficulty with completion of simulated eHealth tasks as evident by taking longer to complete the task were more likely to have more severe COPD as evident by having a greater odds of a CAT score greater than 10 and walking shorter distances in their 6-minute walk tests. Thus, persons with worse COPD—the very patients often targeted with eHealth-related interventions—may require more significant training and infrastructure support, such as greater incorporation of caregivers into the process. Others have made similar recommendations for bridging this digital divide with regard to patients living in rural communities, racial minorities, and persons with low health literacy [19,21-23].

We found that patients who reported owning and using video chat and email, presumably for communicating with friends and family, completed the simulated tasks of submitting answers to CAT items more quickly. With some devices, however, patients who owned the device but reported not using it had worse task times than someone who did not even own the device. Thus, ownership alone may not be a sufficient screening question before an eHealth intervention because owner/nonusers had worse task performance than owners who were more frequent users. Thus, screening questions based on both access to and use of eHealth technologies could serve as surrogates for self-efficacy related to digital health literacy, a component of Health 2.0 skills [26].

In addition to having less advanced COPD, participants who used more and newer communication technologies were also younger and had higher incomes. Accordingly, studies and clinical services that allow for patients to self-select may be composed of relatively healthy patients and thus could make eHealth-related interventions and programs look more effective than studies with a broader sample. Conversely, studies or programs consisting of patients who are older, less affluent, or with more advanced stages of disease may be less effective because these patients are less likely to be comfortable using newer forms of communication. To help ensure effectiveness of eHealth interventions, it may be valuable to design and tailor interventions specific to these persons who are less familiar with technology because older adults, in general, prefer assistance when learning a new technology [20].

Other studies have reported findings suggesting that patients with more severe disease may use technology less often, independent of other demographic factors [27]. This finding has important implications for COPD interventions because patients with certain sociodemographic factors [28] and a history of COPD exacerbations are more likely to experience exacerbations, hospitalizations, and readmissions and therefore are the most in need of intervention. These findings, combined with our finding that demographic factors are associated with lower technology use, might imply that interventions to prevent readmissions in COPD patients may struggle to achieve the desired results based on the interplay between sociodemographics, health history, and experience with technology.

Our findings echo concerns about the relationship between eHealth interventions and health disparities. There was a significant association in most of the analyses between low income, higher disease severity, and lower technology use. Proliferating eHealth initiatives that use advanced technologies could disproportionately benefit wealthier, more native users of technologies, adding to health disparities that already favor those with more financial resources. Although we did not specifically investigate differences between urban and rural patients with COPD, rural patients may have more severe disease than their urban and suburban counterparts [9,29]. This, combined with issues of rural access to high-speed internet, may further exacerbate health disparities for rural COPD patients. Having high-speed internet access would increase someone’s likelihood to have prior experience with newer communication technologies, and their comfort levels with eHealth-related care [21].

This study is subject to several limitations. Although our survey had a high participation rate, we only surveyed and tested participants within a single center in the COPDGene study. Thus, our results may not be generalizable to the population of COPD patients as a whole. Also, unmeasured characteristics may have influenced each participant’s decision to participate in COPDGene and in the eHealth simulation task subsample, factors that presumably could affect their use of the technologies of interest. The majority of our participants were white, and patients belonging to different racial or ethnic groups may have different experiences with technology and barriers to fully participating in eHealth interventions. Also, the survey was only administered in English. Future studies need to investigate the use of technology in diverse populations of COPD patients. Lastly, the cross-sectional nature of this study limits the ability to make causal inferences.

Despite our limitations, we show that patients with COPD have different levels of access and experience using communications technology. With our simulated health tasks, we also showed that older patients and patients with more severe disease had more difficulty using technology. These findings demonstrate the need for education and assistance for patients who are either not as healthy or not as familiar with technology. Testing the effectiveness of new interventions should include assessment of previous technology use and familiarity.

## Appendix

Multimedia Appendix 1 Structured interview script.

Multimedia Appendix 2 Logistic model of variables predicting the odds of a patient having a CAT score ≥10 given age, gender, income, and prior use of the given technology (N=686).

Multimedia Appendix 3 Ordinary least squares model of the relationship between gender, income, and prior use of the given technology and 6-minute walk distance (N=686).

Multimedia Appendix 4 Task time relative to owner/nonuser for nonowner and owner/user adjusted for age, sex, and income.

## Abbreviations

CAT: COPD Assessment Test
COPD: chronic obstructive pulmonary disease
eHealth: electronic health
