# Supplementary material for: The Association Between Technology Use and Health Status in a Chronic Obstructive Pulmonary Disease Cohort: Multi-Method Study
Source: J Med Internet Res. 2018 Apr 2;20(4):e125. doi: 10.2196/jmir.9382 (PMC5902698; doi:10.2196/jmir.9382)
Supplement: Multimedia Appendix 1 [file jmir_v20i4e125_app1.pdf]

Appendix A: Structured interview script

I'm calling to ask a few questions as part of the COPD gene study. Is this an okay time?

(Also administered face-to face after a COPDGene appointment)

The questions are about different technologies people sometimes use to talk to their friends, family, and sometimes their doctors, and nurses. It should take about 5 minutes.

There is no compensation for this part, but we would appreciate your input as we look for ways to make COPD care better. This is voluntary and won't affect anything else you do with the COPD gene study or anything else related to your health care.

Your answers will be confidential, if there are any questions you don't want to answer, let us know and we will move on.

Does this sound like something you would be interested in doing? Or do you have any questions?

If yes…

1. Do you have a cell phone? (Y/N). If answers No skip question #2
    a. Do you carry it with you all the time? (Y/N)
    b. Is it a smartphone (touch screen, run apps?) (Y/N)
2. Do you use text messages? (Y/N)
3. Do you have a computer at home? (Y/N)
4. Do you have a tablet computer like an ipad at home? (Y/N).
5. Do you use email? (Y/N)
6. Do you use a video chat program like Skype or Facetime to talk to family and friends?

That is all of our questions, do you have any questions for me?

Thank you for your time and we look forward to seeing you at your next COPD gene appointment.
