# Supplementary material for: The Association Between Technology Use and Health Status in a Chronic Obstructive Pulmonary Disease Cohort: Multi-Method Study
Source: J Med Internet Res. 2018 Apr 2;20(4):e125. doi: 10.2196/jmir.9382 (PMC5902698; doi:10.2196/jmir.9382)
Supplement: Multimedia Appendix 2 [file jmir_v20i4e125_app2.pdf]

Appendix B: Logistic model of variables predicting the odds of a patient having a CAT score >= 10 given age, gender, income, and prior use of the given technology (N=686).

		Text Messaging	Email	Video Chat
Age		1.02<br>(0.99-2.83, *P* = .19)	1.02<br>(0.99-1.04, *P* = .03)	1.03<br>(1.00-1.05, *P* = .02)
Male		1.05<br>(0.75-1.46, *P* = .79)	1.05<br>(0.75-1.46, *P* = .55)	1.11<br>(0.79-1.56, *P* = .53)
Income				
	<15k	1.58<br>(0.76-3.27, *P* = .21)	1.82<br>(0.85-3.90, *P* = .12)	1.82<br>(0.85-3.89, *P* = .12)
	15-35k	1.00	1.00	1.00
	35-50k	0.54<br>(0.34-0.88, *P* = .01)	0.55<br>(0.34-0.91, *P* = .02)	0.55<br>(0.34-0.91, *P* = .02)
	50-75k	0.46<br>(0.28-0.74, *P* = .001)	0.46<br>(0.28-0.75, *P* = .002)	0.47<br>(0.29-0.77, *P* = .002)
	>75k	0.34<br>(0.20-0.58, *P* < .001)	0.35<br>(0.21-0.60, *P* < .001)	0.36<br>(0.21-0.62, *P* < .001)
	Declined	0.81<br>(0.44-1.52, *P* = .51)	0.88<br>(0.46-1.67, *P* = .69)	0.87<br>(0.46-1.66, *P* = .67)
Technology use				
	Non-Owner	0.58<br>(0.28-1.20, *P* = .13)	0.58<br>(0.28-1.20, *P* = .17)	0.96<br>(0.48-1.93, *P* = .92)
	Non-User	1.00	1.00	1.00
	User	0.67<br>(0.46-0.99, *P* = .04)	0.67<br>(0.46-0.99, *P* = .02)	0.64<br>(0.43-0.93, *P* = .02)

Odds ratios above 1.0 indicate increased odds of having a CAT score greater than or equal to 10 which is indicative of greater disability.
