# Supplementary material for: The Association Between Technology Use and Health Status in a Chronic Obstructive Pulmonary Disease Cohort: Multi-Method Study
Source: J Med Internet Res. 2018 Apr 2;20(4):e125. doi: 10.2196/jmir.9382 (PMC5902698; doi:10.2196/jmir.9382)
Supplement: Multimedia Appendix 3 [file jmir_v20i4e125_app3.pdf]

Appendix C: Ordinary least squares model of the relationship between gender, income, and prior use of the given technology and Six Minute Walk distance (N=686)

		Text Messaging	Email	Video Chat
Age		-14.53 (-18.43 - -10.63, *P* < .001)	-14.83 (-18.44- -11.22, *P* < .001)	-15.27 (-18.87- -11.67, *P* = .001)
Male		87.19 (28.89-145.48, *P* = .003)	85.06 (26.66-143.46, *P* = .004)	82.45 (23.95 – 140.95, *P* = .005)
Income				
	<15k	-190.28 (-320.40 - -60.17, *P* = .004)	-223.68 (-356.27 - -92.10, *P* = .001)	-223.14 (-356.09 - -90.19, *P* = .001)
	15-35k	Reference	Reference	Reference
	35-50k	55.31 (-32.47 – 143.09, *P* = .21)	45.81 (-42.94-134.55, *P* = .30)	47.93 (-41.03 – 136.89, *P* = .28)
	50-75k	78.59 (-7.39 – 164.57, *P* = .07)	78.85 (-7.22-164.91, *P* = .07)	76.75 (-9.72 – 163.21, *P* = .08)
	>75k	135.70 (45.39-226.01, *P* = .003)	129.11 (38.37-219.85, *P* = .005)	126.59 (34.94-218.24, *P* = .006)
	Declined	-2.19 (-116.82-112.43, *P* = .97)	16.80 (-99.11-132.72, *P* = .77)	18.03 (-98.20-134.25, *P* = .76)
Technology use				
	Non-Owner	-36.46 (-166.90-93.98, *P* = .58)	177.83 (18.62-337.03, *P* = .03)	39.83 (-84.23-163.88, *P* = .53)
	Non-User	0	0	0
	User	40.10 (-29.40-109.59, *P* = .25)	177.23 (63.02-192.44, *P* = .002)	79.26 (14.68-143.83, *P* = .01)

Negative values mean shorter distances and more disability
