# Supplementary material for: The Association Between Technology Use and Health Status in a Chronic Obstructive Pulmonary Disease Cohort: Multi-Method Study
Source: J Med Internet Res. 2018 Apr 2;20(4):e125. doi: 10.2196/jmir.9382 (PMC5902698; doi:10.2196/jmir.9382)
Supplement: Multimedia Appendix 4 [file jmir_v20i4e125_app4.pdf]

Appendix D: Task time relative to owner/non-user for non-owner and owner/user adjusted for age, sex and income.

		Task			
(Reference group is owner, non-user)		Laptop	Android	iPad	Phone
Email user					
	Non-owner	+7.7 (*P* = .74)	-6.9 (*P* = .78)	+26.5 (*P* = .55)	+0.5 (*P* = .99)
	User	-48.7 (*P* = .002)	-47.9 (*P* = .003)	+4.5 (*P* = .87)	-0.3 (*P* = .99)
Text user					
	Non-owner	-11.6 (*P* =.24)	-31.5 (*P* = .002)	-1.9 (*P* = .91)	-16.4 (*P* = .10)
	User	-22.6 (*P* = .11)	-29.2 (*P* = .04)	-30.9 (*P* = .19)	-2.2 (*P* = .87)
Video Chat (Skype) user					
	Non-owner	+58.4 (*P* = .002)	+42.7 (*P* = .03)	+22.7 (*P* = .48)	+1.1 (*P* =.96)
	User	-10.2 (*P* = .04)	-6.3 (*P* = .22)	-16.4 (*P* = .06)	-6.3 (*P* = .23)
